# Identification of downstream signaling cascades of ACK1 and prognostic classifiers in non-small cell lung cancer

**DOI:** 10.18632/aging.202408

**Published:** 2021-01-20

**Authors:** Jinhong Zhu, Yang Liu, Meng Zhao, Kui Cao, Jianqun Ma, Shiyun Peng

**Affiliations:** 1 Department of Clinical Laboratory, Biobank, Harbin Medical University Cancer Hospital, Harbin 150040, Heilongjiang, China; 2 Department of Oncology, Harbin Medical University Cancer Hospital, Harbin 150040, Heilongjiang, China; 3 Department of Thoracic Surgery, Harbin Medical University Cancer Hospital, Harbin 150040, Heilongjiang, China; 4 Department of Precision Medicine, Harbin Medical University Cancer Hospital, Harbin 150040, Heilongjiang, China

**Keywords:** ACK1, dasatinib, NSCLC, prognosis, TCGA

## Abstract

*Activated Cdc42-associated kinase 1* (*ACK1*) is an oncogene in multiple cancers, but the underlying mechanisms of its oncogenic role remain unclear in non-small cell lung cancer (NSCLC). Herein, we comprehensively investigated the ACK1-regulated cell processes and downstream signaling pathways, as well as its prognostic value in NSCLC. We found that *ACK1* gene amplification was associated with mRNA levels in The Cancer Genome Atlas (TCGA) lung cancer cohort. The Oncomine databases showed significantly elevated *ACK1* levels in lung cancer. *In vitro*, an ACK1 inhibitor (dasatinib) increased the sensitivity of NSCLC cell lines to AKT or MEK inhibitors. RNA-sequencing results demonstrated that an *ACK1* deficiency in A549 cells affected the MAPK, PI3K/AKT, and Wnt pathways. These results were validated by gene set enrichment analysis (GSEA) of data from 188 lung cancer cell lines. Using Cytoscape, we dissected 14 critical ACK1-regulated genes. The signature with the 14 genes and *ACK1* could significantly dichotomize the TCGA lung cohort regarding overall survival. The prognostic accuracy of this signature was confirmed in five independent lung cancer cohorts and was further validated by a prognostic nomogram. Our study unveiled several downstream signaling pathways for ACK1, and the proposed signature may be a promising prognostic predictor for NSCLC.

## INTRODUCTION

Despite dramatic progress in the understanding of cancer biology and clinical management, lung cancer remains a devastating disease and ranks 1^st^ in both incidence rate and mortality of cancer worldwide [1]. In China, lung cancer is also the 4^th^ most substantial cancer burden [2]. Several factors may help to explain the high mortality of lung cancer, including diagnosis in the late stage, a high propensity for metastasis, and inherent drug resistance. Therefore, it remains imperative to discover efficient diagnostic and prognostic biomarkers and therapeutic targets.

The oncogene *activated Cdc42-associated kinase 1* (*ACK1*), also known as a *nonreceptor tyrosine kinase 2* (*TNK2*), is mapped to chromosome 3q29 and encodes a universally distributed cytoplasmic tyrosine kinase. ACK1 acts as a cytoplasmic effector of activated receptor tyrosine kinases (RTKs). Upon stimulation with extracellular growth factors [heregulin, insulin, epidermal growth factor (EGF), or platelet-derived growth factor (PDGF)], ACK1 can interact with activated transmembrane RTKs and undergo autophosphorylation at Tyr284, consequently conveying extracellular signals to the intracellular effectors [3–5]. ACK1 has been found to participate in the regulation of certain fundamental cellular processes, including proliferation, migration, invasion, and epidermal-mesenchymal transition (EMT) [6].

ACK1 is a multidomain structural protein, comprising tyrosine kinase, SH3, CRIB proline-rich, and ubiquitin-association (Uba) domains [4]. These functional domains confer ACK1 the capacity to bind to a variety of protein molecules and execute complicated functions in terms of the context of a specific milieu [4, 7]. To date, many interacting partners have been identified for ACK1, including clathrin, WW domain-containing oxidoreductase (Wwox), Grb2, EGF receptor (EGFR), AKT1, ubiquitin, androgen receptor, and Nedd4-1/2 E3 ligases [5, 8–12]. ACK1 has been linked to different types of cancer, including prostate cancer [5], ovarian cancer, breast cancer, pancreatic cancer, and lung cancer [13]. ACK1 gene alterations (i.e., amplification, deletion, and mutation) have been detected in various human cancers, ranging from 4% to 27% [4]. The oncogenicity of ACK1 is primarily attributed to its phosphorylation and activation of crucial pro-survival kinases and hormone receptors at different tyrosine residues [3, 4, 12, 14]. ACK1 may phosphorylate AKT at an evolutionarily conserved tyrosine residue at the 176^th^ position (Tyr176) to induce PI3K-independent AKT activation [11]. Moreover, ACK1 phosphorylates androgen receptor (AR) at Tyr267 and Try363 to stimulate the progression of prostate cancers [3]. However, mechanistic studies of ACK1 are very limited and the signaling pathways affected by ACK1 warrant extensive investigation.

Several innovative features can be found in this study. First, we showed that inhibiting ACK1 by dasatinib might sensitize NSCLC cells to MK-2206 (AKT inhibitor) and selumetinib (MEK1/2 inhibitor). Second, by integrating in-house RNA-sequencing (RNA-seq) data and public datasets, we found that ACK1 might regulate the MAPK, PI3K/AKT, and Wnt pathways. Finally, instead of ACK1 alone, we developed a prognostic signature based on ACK1-related genes that can independently predict clinical outcomes in NSCLC.

## RESULTS

### The implication of ACK1 in NSCLC

**Figure 1:**
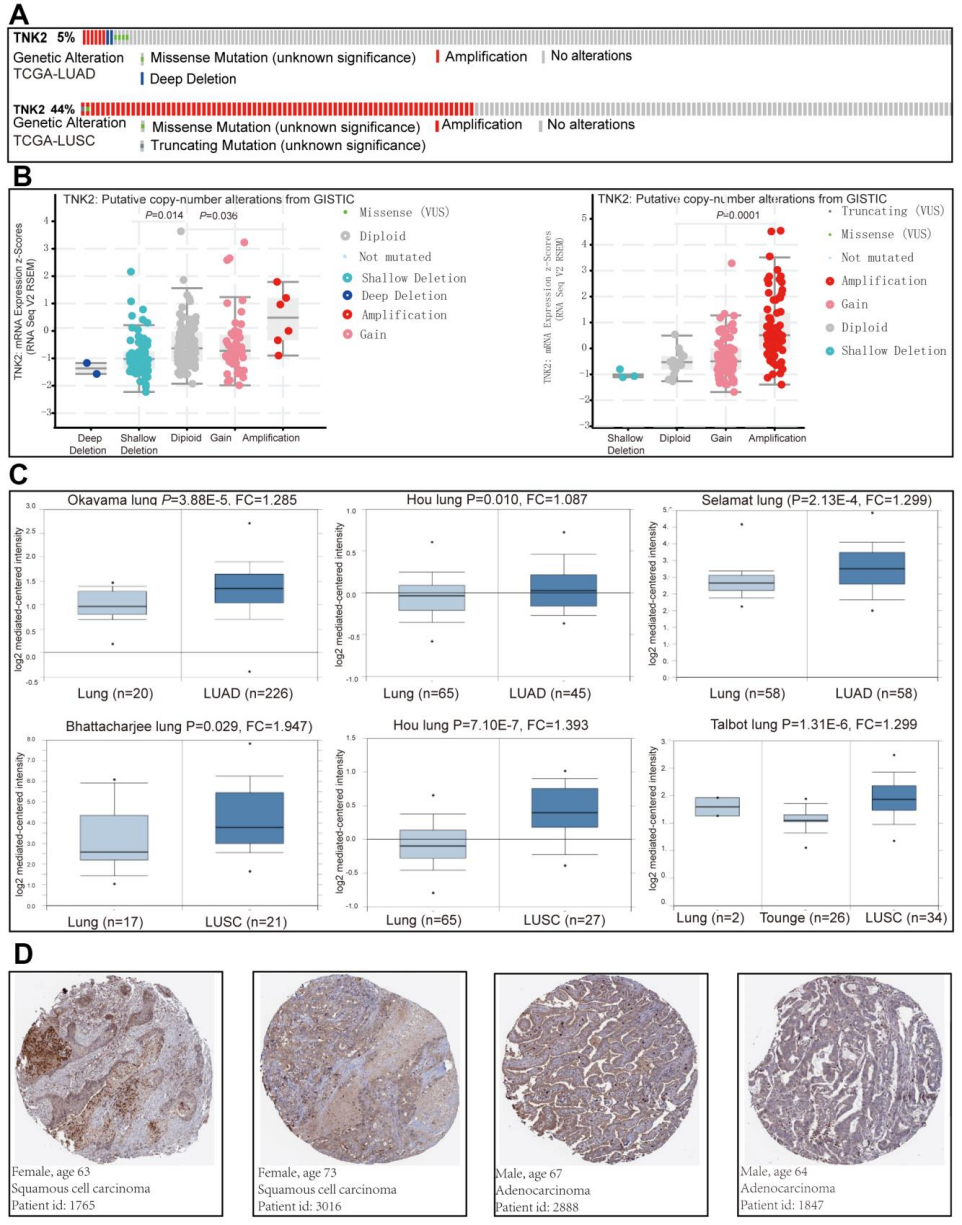
**The implication of ACK1 in NSCLC.** Genetic alterations of the *ACK1* gene in the TCGA-LUAD and TCGA-LUSC cohorts (**A**) (https://www.cbioportal.org). The association between *ACK1* gene copy number and mRNA expression levels (**B**). Significantly elevated mRNA expression levels of the *ACK1* gene in lung cancer in comparison with normal tissues in the independent cohorts from the Oncomine database (**C**). Immunohistochemistry of ACK1 in lung cancer (**D**, Human Protein Atlas). Abbreviation: FC, fold change.

Increasing evidence indicates that ACK1 may be an oncogene involved in various types of cancer. The genetic alterations of ACK1 are displayed in Figure 1A, and *ACK1* amplification was significantly associated with its transcription levels in TCGA lung cancer cohorts (Figure 1B). We also examined *ACK1* gene expression levels in lung cancer tissues and normal tissues using the Oncomine database. Significantly increased ACK1 expression levels were observed in most of the studies (Figure 1C). Figure 1D presents images of immunohistochemical staining of ACK1 in lung cancer tissues (Human Protein Atlas Database).

**Figure 2:**
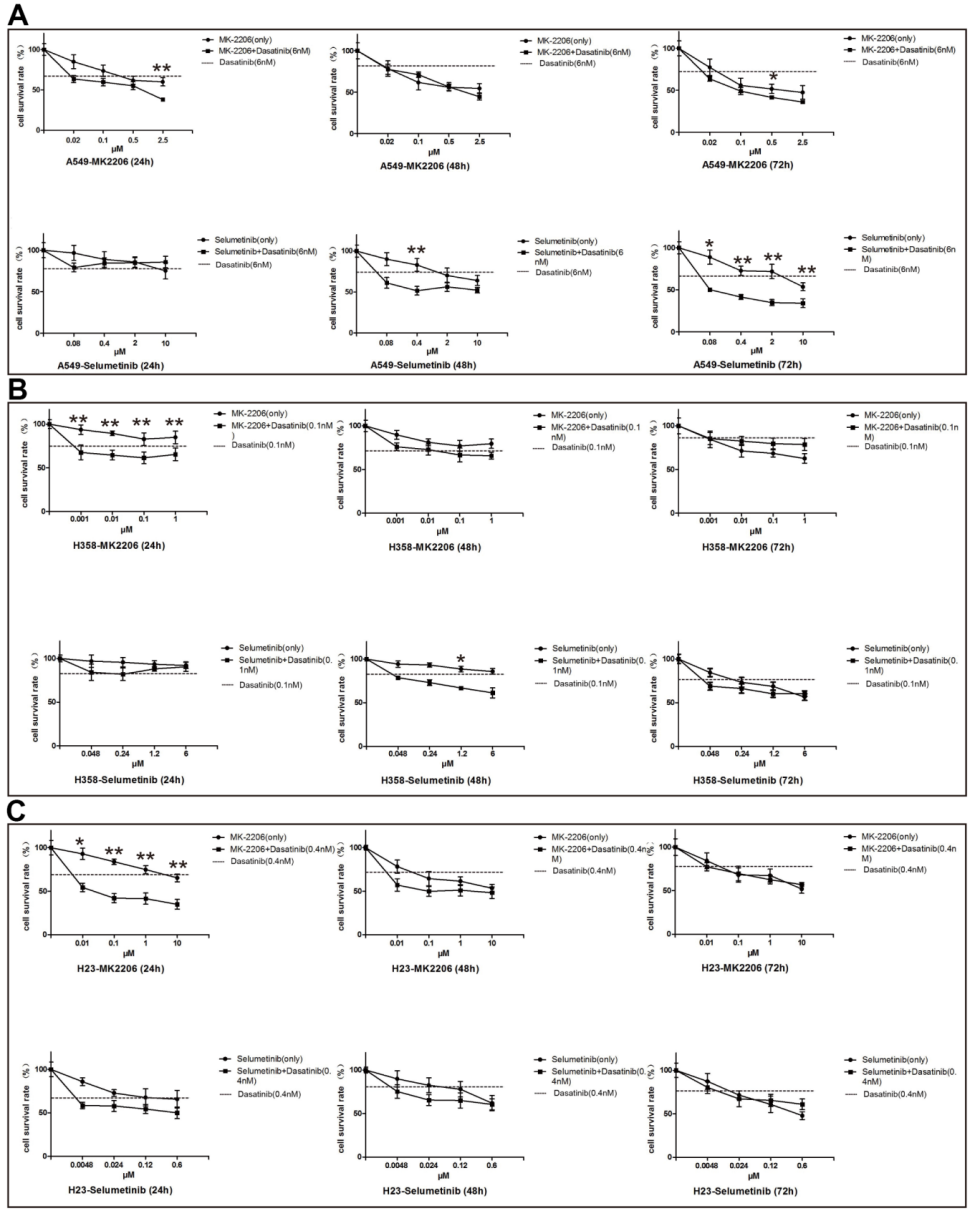
**Inhibitory efficiency of ACK1 inhibitor alone or in combination with MK-2206/selumetinib on NSCLC cell lines.** Proliferation assay of the A549 cell line (**A**), H358 cell line (**B**), and H23 cell line (**C**) treated with drugs as indicated. Combined therapy performed significantly better than single agents in suppressing cell survival. * and ** denoted *P*<0.05 and *P*<0.01, respectively. Data are represented as the mean ± SD.

An *in vitro* study showed that the ACK1 inhibitor, dasatinib could significantly suppress the proliferation of NSCLC A549 cells. The combination of dasatinib and selumetinib (MEK inhibitor) more potently suppressed A549 cell proliferation than either inhibitor alone at 48 and 72 hours after treatment. However, the synergistic inhibition of cell proliferation was observed to a lesser extent for dasatinib and MK-2206 (AKT inhibitor) in the A549 cells (Figure 2A). These drugs were also tested in H23 and H358 cell lines. As shown in Figure 2B, 2C, the efficiency of drugs was cell line-dependent, indicating the importance of precision medicine.

### Identification of differentially expressed genes (DEGs) with RNA-seq after silencing of *ACK1* gene

**Figure 3:**
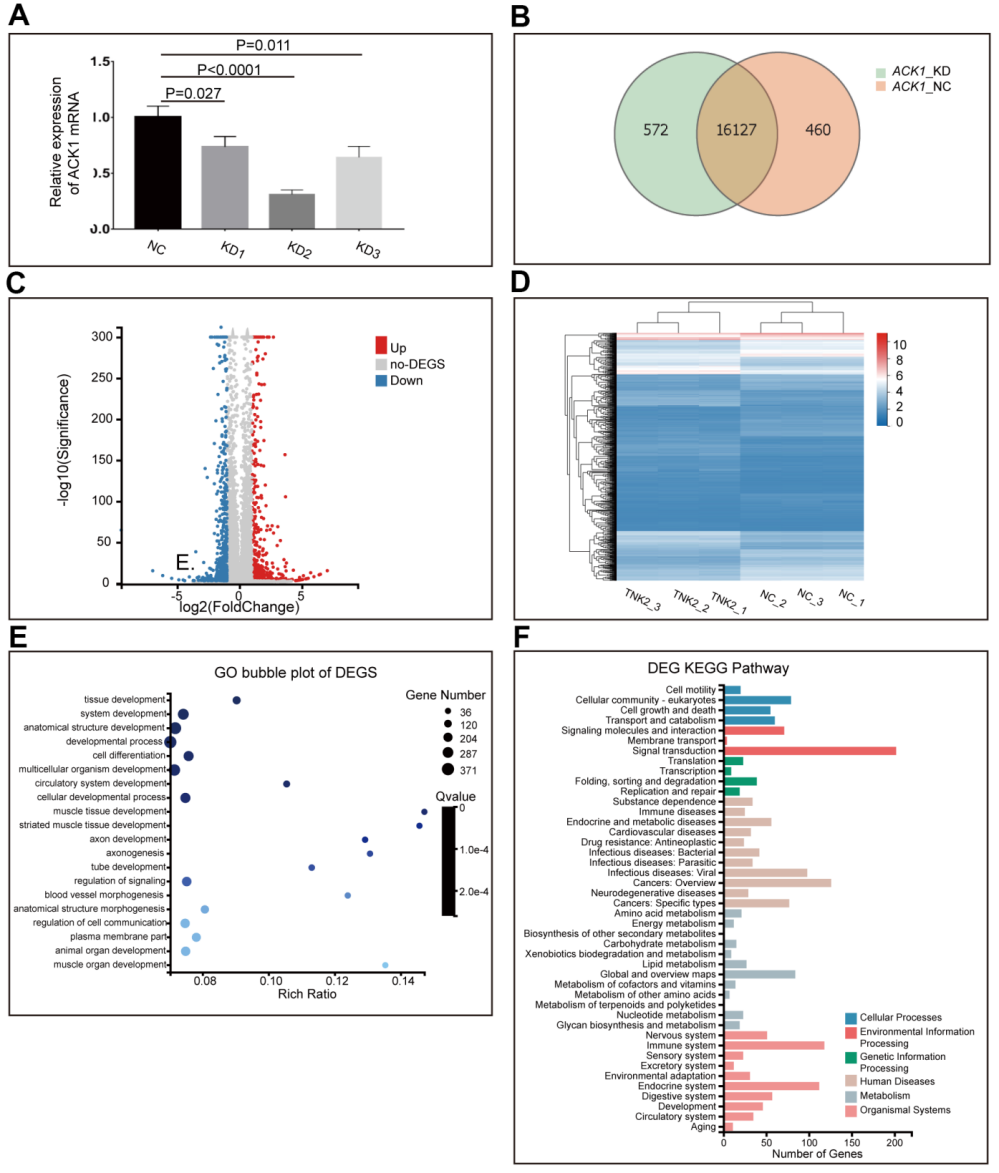
**Knockdown (KD) of *ACK1/TNK2* in A549 cells, followed by RNA-seq.** *ACK1* was silenced using three lentivirus-mediated shRNAs (**A**). The shRNA showing the highest efficiency of the *ACK1* gene knockdown was used for subsequent experiments. Overlapping genes were identified in the negative control (NC) and KD groups (**B**). The volcano plot (**C**) indicated the significantly up- and downregulated genes after the silencing of *ACK1* [absolute value of log2 (fold change) ≥1, *P*<0.001]. Based on differentially expressed genes (DEGs), three NC and three KD samples (sh*ACK1*/*TNK2*) were well clustered (**D**). Gene Ontology enrichment analysis of DEGs (**E**). KEGG pathway annotation of DEGs (**F**).

Results from other teams and ours have shown the suppression of NSCLC cell proliferation by diverse ACK1 inhibitors. However, the downstream signaling cascades of ACK1 remain mostly unknown. In this study, we knocked down the *ACK1* gene in A549 cells with the lentivirus delivery system (Figure 3A) and checked the affected downstream signaling pathways. The lentiviruses carrying shRNA-KD2 exhibited the highest knockdown efficiency and were used to infect A549 cells for RNA-seq. Approximately 21.78 M data were generated for each of 6 samples on average using the BGISEQ-500 platform. In total, 17,159 genes were detected in 6 samples, among which 16,127 were found in both *ACK1-*negative control (NC) and *ACK1*-knockdown (KD) samples (Figure 3B). After analysis of our RNA-seq data following a previously published method [15], we finally obtained 1,076 differentially expressed genes (DEGs) (fold change ≥2 and adjusted *P* value <0.001). A volcano plot (Figure 3C) depicts the distribution of DEGs, and a heatmap (Figure 3D) indicates that DEGs could properly cluster samples into NC and KD groups.

### Gene Ontology (GO) and Kyoto Encyclopedia of Genes and Genomes (KEGG) enrichment analyses of DEGs

DEGs were subjected to GO enrichment analysis and KEGG pathway annotation. DEGs were significantly enriched in tissue development, developmental process, cell differentiation, cellular developmental process, and regulation of signaling (Figure 3E). KEGG pathway annotation revealed that DEGs were mainly distributed in signaling molecules and interactions, signal transduction, cancers: overview, cancers: specific types, immune system, and endocrine system (Figure 3F).

### Protein-protein interaction (PPI) network construction and module analysis

We first constructed a PPI network of DEGs using the Search Tool for the Retrieval of Interacting Genes (STRING) database with a minimum required interaction score. The tightly assembled network suggested that these DEGs were biologically functionally connected, but not randomly scattered (data not shown). The resulting data describing the coordinates of nodes in the network were imported into Cytoscape (version 3.4.0) for further analysis.

### Hub gene selection and analysis

**Figure 4:**
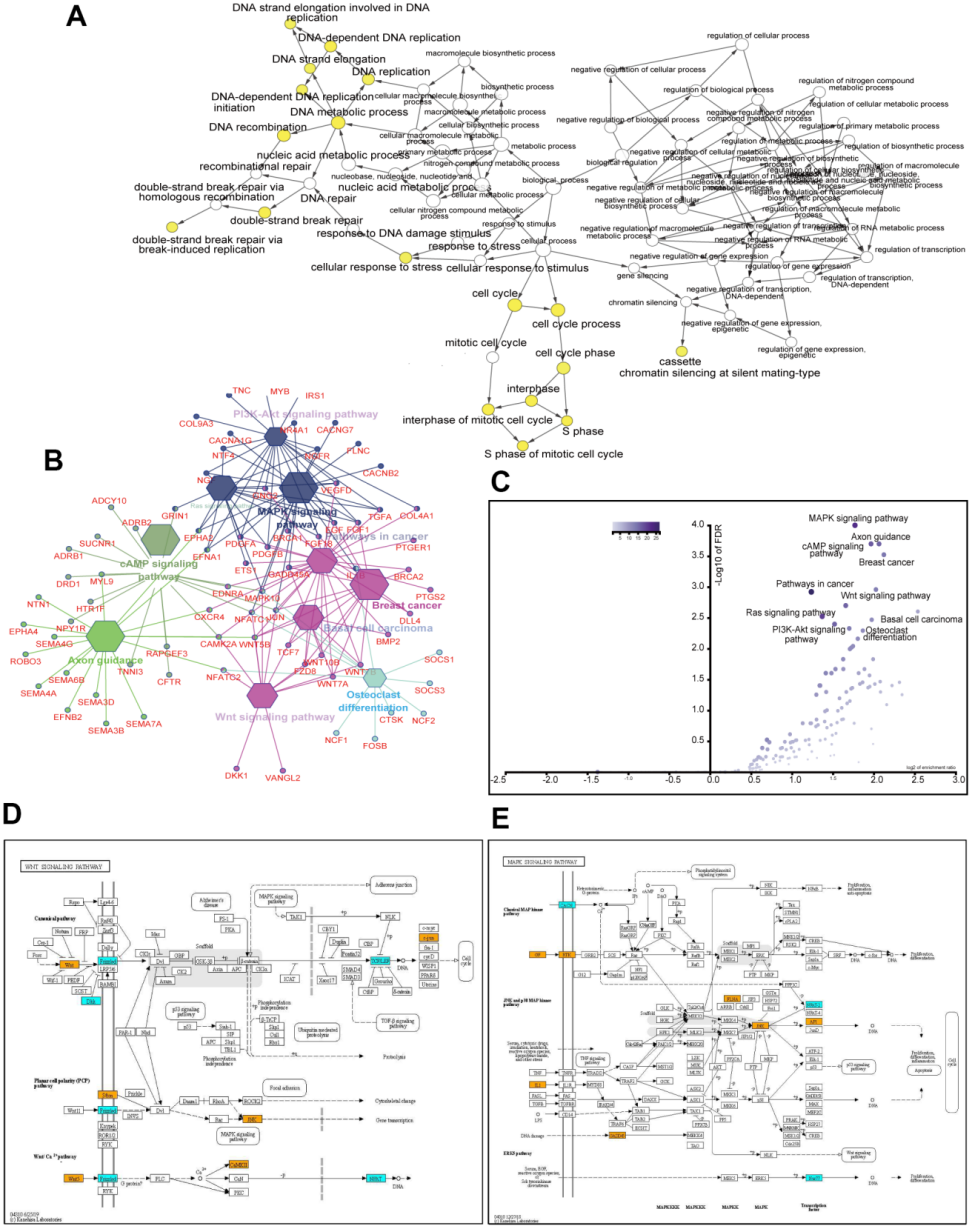
**Analysis of 219 hub genes with degree ≥10.** GO biological process analysis using the BinGo plug-in of Cytoscape (**A**). KEGG pathway analysis using the ClueGo plug-in of Cytoscape (**B**). Volcano plot of the enriched pathways with *P* values and enrichment scores (**C**). Altered genes in the Wnt signaling pathway (**D**). Altered genes in the MAPK signaling pathway (**E**). Orange and cyan rectangles indicate the upregulation and downregulation of genes, respectively, after the *ACK1* gene knockdown. Only DEGs (fold change ≥2 and adjusted *P* value <0.001) are colored.

Using the CytoHubba Cytoscape plug-in, we identified 219 hub genes with degrees ≥10, 107 genes with degrees ≥15, and 57 genes with degrees ≥20. We analyzed the biological processes of 219 hub genes using the Cytoscape plug-in, Biological Networks Gene Ontology tool (BiNGO) (version 3.0.3). As shown in Figure 4A, the yellow spots, in which genes were mostly enriched, indicated that these genes were involved primarily in DNA replication, DNA repair, cell cycle, and cellular response to stress. Another ClueGo plug-in of Cytoscape revealed that these genes mainly fell into malignancy-related pathways, including the MAPK, cAMP, Wnt, and PI3K-Akt signaling pathways, pathways in cancer, and axon guidance (Figure 4B). The enriched scores and -log10 of FDR are shown in Figure 4C. Hub genes enriched in Wnt and MAPK signaling pathways are visualized in Figure 4D, 4E, respectively.

**Figure 5:**
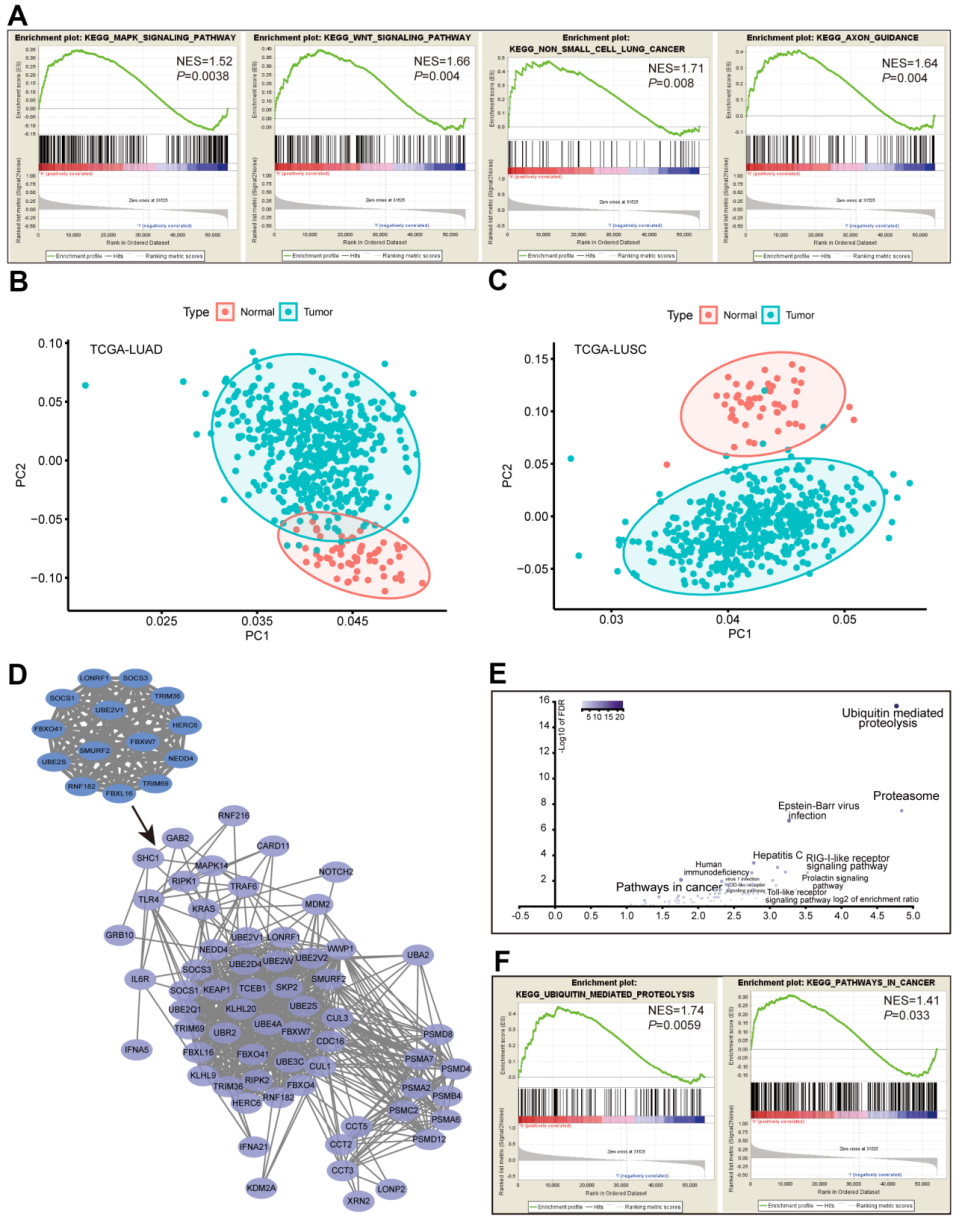
**Analysis of the ACK1 signaling pathways.** RNA-seq data of 188 lung cancer cell lines were retrieved from the CCLE database. GSEA was performed after dividing cell lines into *ACK1*^high^ and *ACK1*^low^ groups by the average *ACK1* expression level (**A**). Principal component analysis (PCA) of the 57 hub genes (degree≥20) was carried out in the TCGA-LUAD (**B**) and TCGA-LUSC (**C**) versus normal tissues. The most significant 14-gene module was derived from DEGs, which captured 50 coexpressed genes in the TCGA-LUAD cohort (**D**). Most of the 64 genes were enriched in the ubiquitin-mediated proteolysis and the proteasome (**E**), which was validated by GSEA using CCLE lung cancer cell data (**F**).

In addition, we retrieved RNA-seq data for 188 lung cancer cell lines from the Cancer Cell Line Encyclopedia (CCLE) database and dichotomized the cancer cell lines by the average expression levels of the *ACK1*. Gene set enrichment analysis (GSEA) performed between the *ACK1*^high^ and *ACK1*^low^ groups confirmed the enrichment of ACK1-regulated genes in the MAPK, Wnt, NSCLC, and axon guidance pathways (Figure 5A). Heatmaps were plotted to depict the mRNA expression profile of 57 hub genes with degrees ≥20 in LUAD and LUSC samples compared with the respective normal tissues (Supplementary Figure 1). We further carried out principal component analysis (PCA), a dimension reduction method, to compare the mRNA expression profiles of the 57 hub genes between lung cancer and normal tumor tissues. PCA could discriminate tumor samples from normal tissues in the TCGA-LUAD (Figure 5B) and TCGA-LUSC (Figure 5C) cohorts. These results suggest that these differentially expressed hub genes are adequate to define tumor samples.

### Detection of the most significant molecular complex

Using the Molecular Complex Detection (MCODE) plug-in of Cytoscape, we identified the most significant module composed of 14 nodes. The names, abbreviations, and functions of the node genes are shown in Table 1. More than half of the node genes are involved in the ubiquitination process. The 14 essential genes captured an extra 50 tightly co-expressed genes in TCGA-LUAD (https://www.cbioportal.org), as shown in Figure 5D. KEGG enrichment analysis revealed that these 64 genes mainly related to ubiquitin-mediated proteolysis, proteasome, and pathways in cancer (Figure 5E). GSEA analysis of the CCLE database further validated that ACK1 was associated with ubiquitin-associated proteolysis and pathways in cancer in lung cancer cells (Figure 5F).

**Table 1:** Functions of 14 key genes in the most significant module.

**No.**	**Gene symbol**	**Full name**	**Function**
1	SOCS3	suppressor of cytokine signaling 3	A suppressor of cytokine signaling family.
2	SOCS1	suppressor of cytokine signaling 1	A suppressor of cytokine signaling family.
3	TRIM36	tripartite motif containing 36	a member of the tripartite motif (TRIM) family, consisting three zinc-binding domains, a RING, a B-box type 1 and a B-box type 2, and a coiled-coil region.
4	TRIM69	tripartite motif containing 69	
5	SMURF2	SMURF2	SMAD specific E3 ubiquitin protein ligase 2
6	LONRF1	LONRF1	LON peptidase N-terminal domain and ring finger 1
7	HERC6	HERC6	HECT and RLD domain containing E3 ubiquitin protein ligase family member 6
8	RNF182	RNF182	ring finger protein 182
9	FBXW7	FBXW7	E3 ubiquitin protein ligase
10	UBE2V1	ubiquitin-conjugating enzyme E2 variant 1	It can cause transcriptional activation of the human FOS proto-oncogene.
11	UBE2S	ubiquitin-conjugating enzyme E2S	A member of the ubiquitin-conjugating enzyme family.
12	FBXL16	F-box and leucine-rich repeat protein 16	F-box proteins interact with ubiquitination targets
13	NEDD4	NEDD4	E3 ubiquitin protein ligase
14	FBXO41	F-box protein 41	Involved in phosphorylation-dependent ubiquitination.

### Prognostic values of gene signature in the most significant module

**Figure 6:**
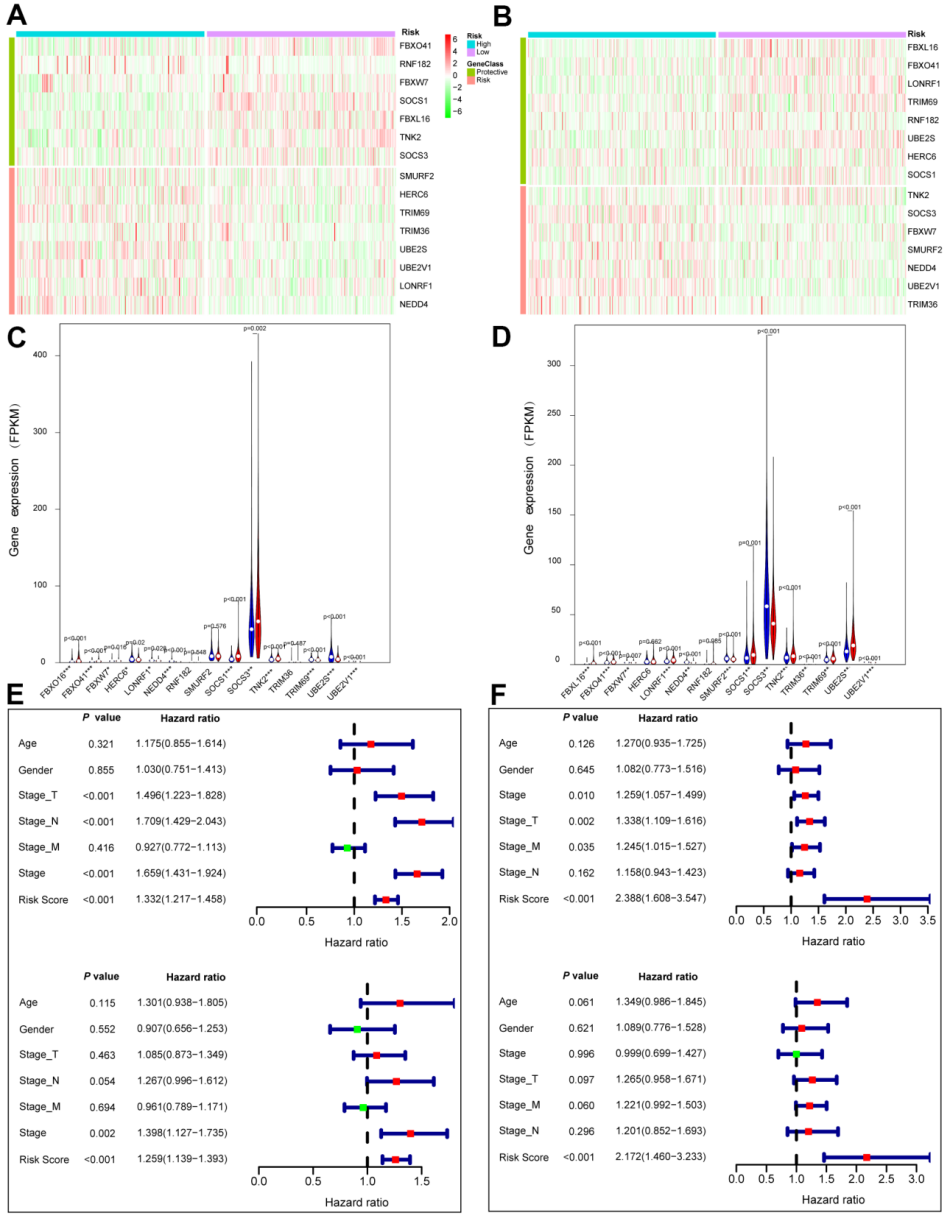
**Prognostic values of the gene signature comprising *ACK1* and the 14 genes of the most significant module in the TCGA lung cancer patients.** Patients were classified into low (green) and high (red) risk groups according to risk scores. Heatmaps of 15 gene expression profiles in the low and high risk LUAD (**A**) and LUSC (**B**) patients. Comparison of the 15 gene expression levels between high (blue) and low (red) risk groups in LUAD (**C**) and LUAD (**D**). Univariate (upper panel) and multivariate (lower panel) Cox regression analyses in LUAD (**E**) and LUSC (**F**).

*ACK1*/*TNK2* alone was not sufficient to significantly stratify patients according to clinical outcomes in LUAD and LUSC (Supplementary Figure 2). We wondered whether the combination of *ACK1*/*TNK2* and the 14 key genes can improve prognostic prediction. A total of 490 LUAD and 488 LUSC patients with survival data were obtained from the TCGA project. The association of the 15 genes with lung cancer survival was first evaluated by univariate Cox regression analysis. Genes with a hazard ratio (HR) <1 or >1 were viewed as protective or risk genes, respectively. The risk score was calculated for each case based on the expression of each gene and its correlation with survival. The patients were divided into high- and low-risk groups with the median risk score as the cutoff value. In the heatmaps of gene expression, the risk genes were preferentially expressed in the high-risk groups and vice versa in both the LUAD (Figure 6A) and LUSC (Figure 6B) cohorts. A comparison of mRNA expression of individual genes between two groups was conducted for LUAD (Figure 6C) and LUSC (Figure 6D).

**Figure 7:**
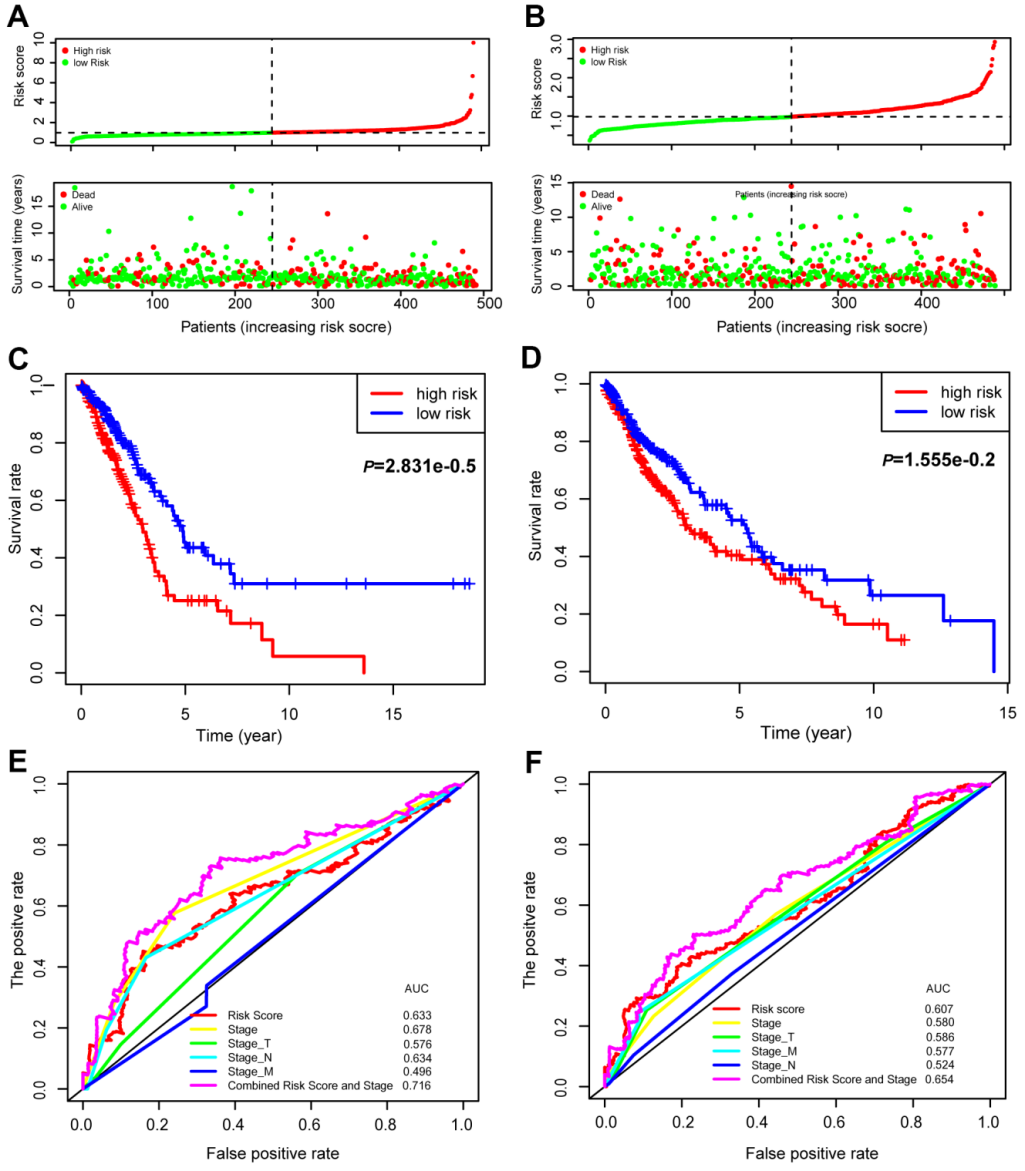
**Prognostic values of the 15-gene signature in the TCGA lung cancer patients.** The distribution of risk scores and survival statuses of patients in the LUAD (**A**) and LUSC cohorts (**B**). Kaplan-Meier survival curves of patients defined by low and high risk scores in LUAD (**C**) and LUSC (**D**). ROC curves with different characteristics of patients, as indicated in LUAD (**E**) and LUSC (**F**).

**Figure 8:**
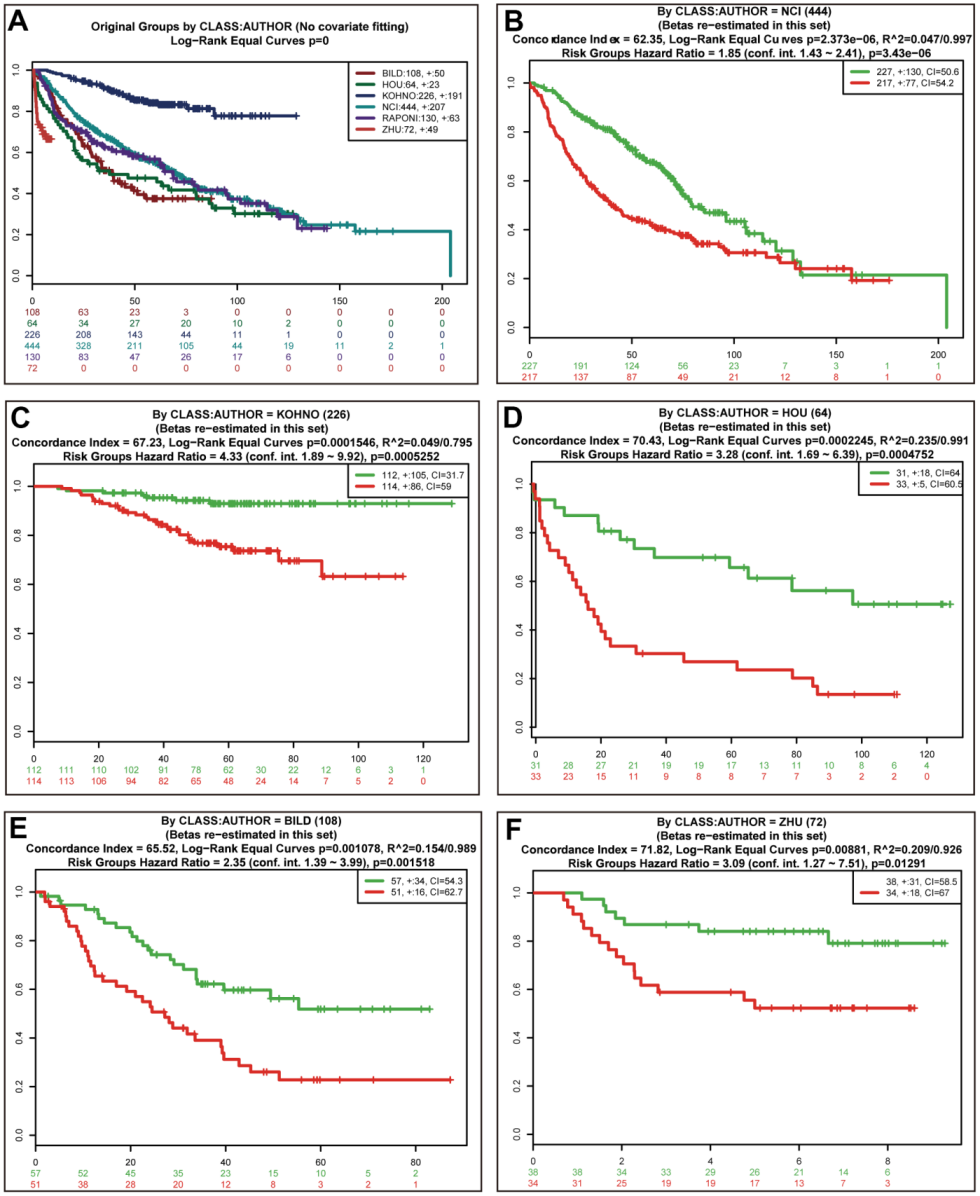
**Validation of the prognostic power of risk scores in the independent lung cancer cohorts.** Kaplan-Meier survival curves of 6 independent lung cancer cohorts (**A**). Performance of risk scores in the NCI (**B**), KOHNO (**C**), HOU (**D**), BILD (**E**), and ZHU (**F**) cohorts.

Univariate (upper panel) and multivariate (lower panel) Cox regression analyses were conducted in LUAD (Figure 6E). The risk score was an independent prognostic predictor in LUAD [hazard ratio (HR)=1.259, 95% confidence interval (CI)=1.139-1.393]. Similar results were observed for LUSC (Figure 6F). The distribution of the risk scores and survival statuses of patients is exhibited for LUAD (Figure 7A) and LUSC (Figure 7B). The risk scores successfully defined LUAD (Figure 7C, log-rank test, *P*<0.0001) and LUSC (Figure 7D, log-rank test, *P*=0.0156) into subgroups with significantly different survival rates. Receiver operating characteristic (ROC) curves were adopted to determine the predictive efficiency of the prognostic signature. The areas under the curve (AUC) of the risk score were 0.633 and 0.607 for LUAD (Figure 7E) and LUSC (Figure 7F), respectively. The combination of risk score and stage could achieve a better prognostic accuracy than either factor alone, and the combined AUCs were 0.716 in LUAD and 0.654 in LUSC. We also tested the predictive power of this prognostic classifier in six independent lung cancer cohorts [16]. The gene signature successfully classified lung cancer patients into subgroups with significantly distinct survival for the NCI (HR=1.85, 95% CI=1.43-2.41, *P*<0.0001), KOHNO (HR=4.33, 95% CI=1.89-9.92, *P*<0.001), Hou (HR=3.28, 95% CI=1.69-6.39, *P*<0.001), BILD (HR=2.35, 95% CI=1.39-3.99, *P*<0.01), and Zhu (HR=3.09, 95% CI=1.27-7.51, *P*=0.013) cohorts, except for the PAPONI cohort (Figure 8).

### Construction of a nomogram

**Figure 9:**
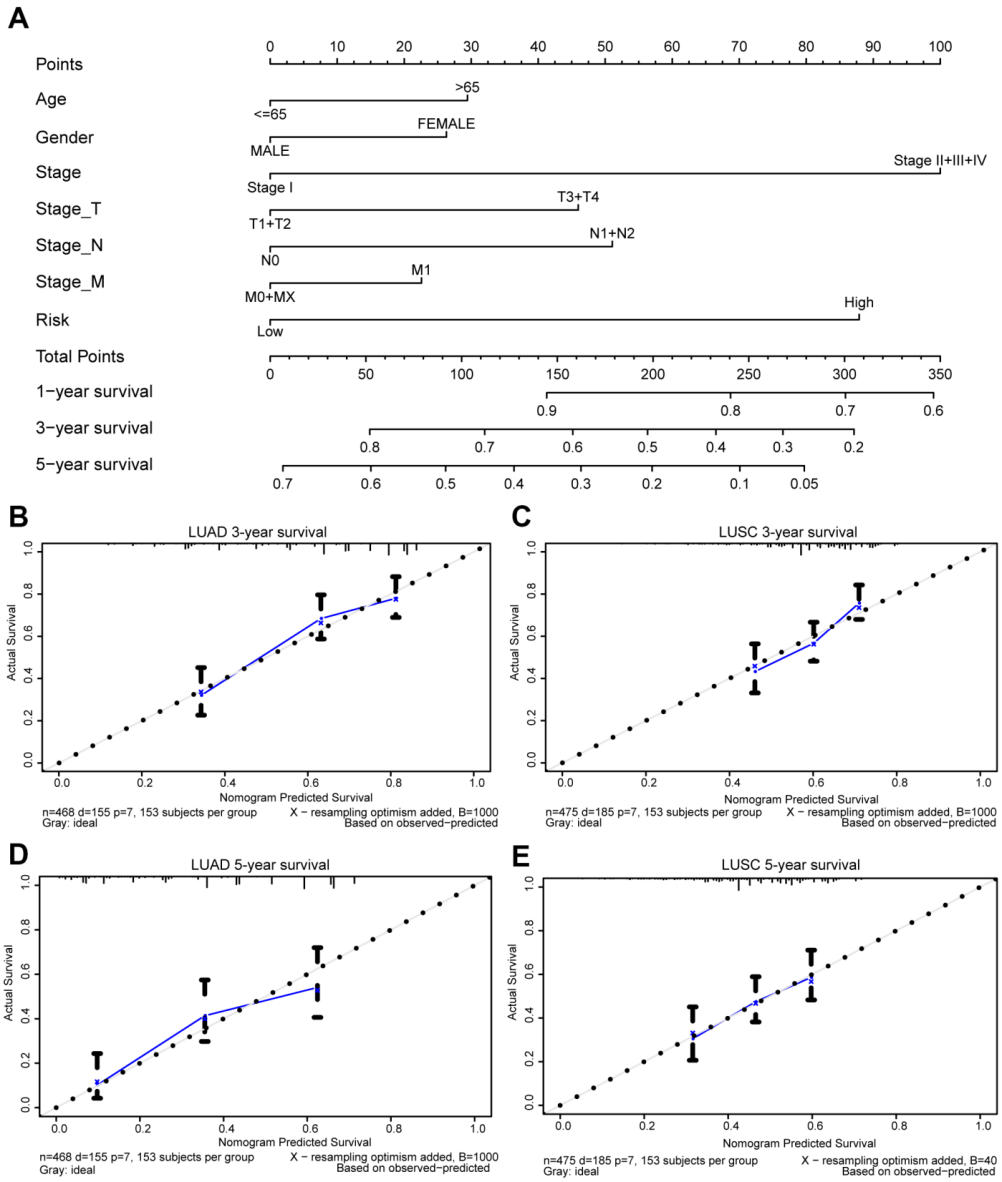
**Prognostic nomogram for TCGA lung cancer cohorts.** Nomogram for evaluating the survival probability of TCGA-LUAD patients (**A**). The calibration curves for predicting patient survival in TCGA-LUAD (**B**, **D**) and TCGA-LUSC (**C**, **E**). Overall survival (OS) derived from the nomogram is plotted on the x-axis, and actual OS is displayed on the y-axis. A plot approaching the 45° dashed line would show an ideal calibration model indicating the perfect concordance between the predicted probabilities and the actual survival.

Moreover, we generated a nomogram to assess the performance of the risk score in combination with the clinical characteristics of NSCLC patients in predicting the prognosis (Figure 9A). The concordance index (*c*-index) values, which measure the level of agreement between predicted probabilities and the actual survival statuses, were 0.7 and 0.6 for LUAD and LUSC, respectively. The calibration curves for 3- and 5-year survival were also plotted for LUAD and LUSC (Figure 9B–9E).

## DISCUSSION

ACK1 is a nonreceptor tyrosine kinase, the deregulation of which may drive hallmarks of cancer, including cell proliferation, migration/metastasis, and EMT. The oncogenicity of ACK1 is mostly due to its phosphorylation and activation of crucial pro-survival kinases and hormone receptors at different tyrosine residues, as well as inactivation of tumor suppressors in cancer cells.

The implications of ACK1 in lung cancer were first reported decades ago. A relatively high frequency of *ACK1* amplification in primary lung cancer was observed, coincident with augmented *ACK1* mRNA levels [13]. ACK1 overexpression was more frequently detected in late-stage than in early-stage tumors. However, the oncogenic and prognostic roles of ACK1 in lung cancer warrant in-depth investigation. In this study, we validated *ACK1* amplification and the association between *ACK1* mRNA expression and copy number variation. The independent lung cancer cohort further verified significantly increased *ACK1* expression levels in cancer tissue compared with those in normal tissue.

ACK1 has been considered a novel therapeutic cancer target. By screening 1,447 available drugs for their abilities against ACK1, Phatak et al. found that dasatinib directly bonded to ACK1 and inhibited its activity at a half-maximal inhibitory concentration (IC50) concentration as low as 1 nm [17]. However, the efficiency of dasatinib as a single agent was unsatisfactory in a clinical phase study [18]. Because cancer is a complex disease that frequently results from the deregulation of different signaling pathways, combined therapies may be a better strategy than single agents. Cubitt et al. generated a system to test the synergistic effects of various combination therapies in several sarcoma cell lines with targeted and cytotoxic drugs [19]. Dasatinib in combination with MK-2206 (AKT inhibitor) outperformed single agents in the suppression of cell proliferation. The combination of saracatinib (Src inhibitor) and selumetinib (MEK-1/2 inhibitor) was also tested, but showed no promise [19]. Inhibition of ACK1 reduced the migration and invasion of KRAS mutant lung adenocarcinoma [20]. Moreover, ACK1 stabilizes EGFR, and knockdown of *ACK1* increases the sensitivity of renal carcinoma cells to gefitinib [6]. In the current study, we tested the combination of dasatinib with either MK-2206 or selumetinib in three NSCLC cell lines. We found that combination therapy did show promise in inhibiting cancer cell proliferation, but the effects were time-, dosage-, and cell line-dependent. Recently, dasatinib was shown to enhance the sensitivity of KRAS mutant cells to trametinib (MEK1/MEK2 inhibitor) in different cancer types, including H23 and H358 NSCLC cell lines. Mechanistic studies revealed that dasatinib-mediated inhibition of YAP/TAZ signaling might be responsible for the synergistic inhibitory effects of these two agents on cancer cells [21]. However, the underlying mechanisms, especially downstream signal cascades affected by ACK1, remain largely unclarified.

We next attempted to scrutinize the signaling pathways impacted by the silencing of *ACK1* in A549 cells using RNA-seq. Our KEGG enrichment analysis identified several signaling pathways under the regulation of ACK1 in NSCLC, including the MAPK, cAMP, Wnt, and PI3K-Akt signaling pathways. These results were supported by our GSEA analysis of the RNA-seq data of 188 lung cancer cell lines downloaded from the CCLE database. Consistent with our finding, silencing of *ACK1* inhibited the phosphorylation of ERK and AKT (Ser473), as well as the proliferation of renal cancer-derived cells, and reversed the EMT [6].

In mouse embryonic fibroblasts (MEFs), normal prostate cells, and MCF-7 cells, in response to EGF stimuli, ACK1 directly interacted with AKT and phosphorylated the latter at Tyr176 in the kinase domain [11]. Phosphorylated AKT moved to the plasma membrane and was further phosphorylated at Ser473. Conversely, knockdown of *ACK1* resulted in a decreased Ser473 phosphorylation of AKT [11]. It was further demonstrated that activation of the RTK/ACK1/AKT pathway promotes the trafficking of both endogenous pTyr284-ACK1 and pTyr176-AKT to the nucleus [11]. Nuclear pTyr176-AKT enhanced cell cycle progression and inhibited apoptosis by phosphorylating FoxO transcription factors and subsequently inhibiting transcriptional activation of target genes (e.g., *p21*, *p27KIP1*, and *Bim-1*) [11, 22]. Breast cancer patients with high expression levels of Tyr176-phosphorylated AKT and Tyr284-phosphorylated ACK1 were significantly more likely to have unfavorable outcomes [11]. ACK1 was also shown to promote the phosphorylation and nuclear localization of STAT3 in cultured human embryonic kidney HEK293T cells and the positive correlation between ACK1 and the levels of tyrosine-phosphorylated STAT3 was validated in primary lung adenocarcinoma (ADC) cells [23]. Intriguingly, many DEGs were also enriched in axon guidance in the current study. Although the pathological relevance of the neural signaling is not clear in NSCLC, ACK1 is indeed highly expressed in the brain [24]. ACK1 plays a role in neurotrophin signaling. In the developing brain, once activated by neurotrophins, ACK1 sequentially triggers AKT phosphorylation to fuel cell proliferation and migration [25].

MCODE was used to extract the most significant module from the complicated network of 1,076 DEGs. The majority of molecules in this module are related to ubiquitination, which was also evidenced by our results derived from the CCLE. Consistent with this finding, several studies revealed the implication of ACK1 in the ubiquitination process [6, 8, 26]. ACK1 possesses a ubiquitin-associated (Uba) domain at the carboxyl terminus, via which ACK1 binds to ubiquitins in both poly- and mono-forms. ACK1 regulates EGFR degradation [8]. Upon EGF stimuli, phosphorylated EGFR at the tyrosine residue forms a complex with ACK1 and cotranslocated into EEA-1-positive vesicles [8]. This interaction facilitated ACK1 to mediate EGFR degradation through the Uba domain [8]. Moreover, the involvement of HECT E3 ubiquitin ligases Nedd4-1 and Nedd4-2 in ACK1 ubiquitination and degradation was also demonstrated [9, 10]. Moreover, ACK1 amplification was shown to contribute to cell proliferation and colony formation in gastric tumorigenesis by boosting the ubiquitination and degradation of p53 [26].

Moreover, the prognostic value of ACK1 has also evoked attention. Hu et al. reported that the high expression level of ACK1 was significantly associated with poor survival in NSCLC. Tan et al. found that ACK1 expression levels were significantly elevated in lung adenocarcinoma compared with nontumor tissues [20] Intriguingly, in 210 Singaporean lung adenocarcinomas, ACK1 expression in the adjacent nontumor tissues, but not in the tumors, was an independent predictor of prognosis [20]. Additionally, the association between ACK1 and survival also failed to be established in the TCGA LUAD and LUSC cohorts. The discrepancies in survival results across studies may result from differences in sample size, sampling, immunohistochemistry staining conditions, and quantitative methods. Taken together, these results suggest that ACK1 alone may not be a potent predictor of prognosis in NSCLC. On the other hand, several studies have demonstrated that the expression signature of a panel of relevant genes might be able to serve as prognostic classifiers in cancer [27–31]. In this study, we found that *ACK1*, in combination with 14 DEGs in the most significant module, could define TCGA-LUAD into two subgroups with significantly different survival. A similar prognostic potential of these genes was observed in TCGA-LUSC. The prognostic value of this signature of 15 genes was further validated in five independent lung cancer study populations, as well as in the prognostic nomogram.

Several limitations of the study should be noted. First, *in vitro* and *in vivo* evidence should be provided in the future to support the implication of ACK1 in NSCLC. Second, due to the retrospective design, the results of this study should be explained cautiously. Third, the predictive accuracy of the prognostic model should be validated in different NSCLC cohorts before it can be applied in the clinical setting.

## CONCLUSIONS

Our results suggest that combination therapy based on the inhibition of ACK1 can suppress lung cancer cell proliferation in a cell line-dependent manner. MAPK, Wnt, and PI3K-AKT may be under the control of ACK1 in lung cancer and ACK1 may partially regulate target proteins by facilitating the ubiquitination process. Finally, a prognostic gene signature can be developed with the *ACK1* and related genes.

## MATERIALS AND METHODS

NSCLC cell lines A549, H23, and H358 were maintained in Dulbecco’s modified Eagle’s medium (Gibco, Thermo Fisher Scientific, USA) supplemented with 1% non-essential amino acids (Gibco, Thermo Fisher Scientific, USA) and 5% fetal calf serum (FCS) (Gibco, Thermo Fisher Scientific, USA). Moreover, 10 units/ml of penicillin-G and 10 mg/ml streptomycin were added to the medium to combat contamination. Cells were grown under common conditions with 5% carbon dioxide at 37° C.

### Drug sensitivity assay

A549 cells were seeded in 96-well plates at a density of 2x10^3^/well and maintained in an incubator overnight. After the removal of the culturing medium, and the cells were fed with fresh medium containing dasatinib, selumetinib, and MK-2206 (MedChemExpress LLC. Monmouth Junction, USA) alone, or in combination. Cell viability was measured by the Dojindo cell counting kit-8 (CCK-8, GlpBio, USA) at 24, 48, and 72 hours.

### ACK1 knockdown and quantitation in NSCLC A549 cell line

To silence *ACK1* expression in the A549 cell line, lentivirus carrying *ACK1*/*TNK2*-RNAi (tgCTTCCTCTTCCACCCAATT, GeneChem, Shanghai, China) was introduced into the A549 cells (*ACK1*-KD). Empty lentivirus vectors were used to infect an extra set of A549 cells as a negative control (NC). Upon reaching 90% confluence in the flasks, cells were washed with PBS and harvested with TRIzol reagent (Invitrogen, Thermo Fisher Scientific, USA). Total RNA was extracted following the manufacturer’s manual. *ACK1* transcript levels in *ACK1*-KD and NC cells were examined using real-time PCR. The primer pair used to amplify the human *ACK1* gene is given as follows: forward primer, 5’- AGCCTCACCTGCCTCATTG -3’, and reverse primer, 5’- GCACTTCACAGCCACACTC -3’. Additionally, a fraction of GAPDH was amplified by PCR as an internal control with the following primers: forward primer, 5’- TGACTTCAACAGCGACACCCA -3’, and reverse primer, 5’- CACCCTGTTGCTGTAGCCAAA-3’.

### rRNA depletion and transcriptome sequencing

The StepOnePlus System (Applied Biosystems, Thermo Fisher Scientific, USA) and the Agilent 2100 Bioanalyzer (Agilent Technologies, USA) were used to measure the amount and integrity of the sample libraries for quality control. The integrity of RNA samples was determined by the values of the RNA integrity number (RIN). The average RIN is 9.52 for these samples.

The RNAiso Plus Kit (TAKARA, Japan) was adopted to purify the total RNA from *ACK1*-NC and *ACK1*-KD A549 cells followed by DNase I digestion. rRNAs were eliminated from the total RNA using the RiboMinus Eukaryote Kit (Qiagen, USA) to obtain pure mRNA. The resulting RNAs were further shred using Ambion Fragmentation Solution (Thermo Fisher Scientific, USA). The mRNA pieces were used as a template to produce cDNAs. Finally, the BGISEQ-500 platform was used to carry out RNA sequencing for six samples. The DEGs between the *ACK1*-NC and *ACK1*-KD groups were determined (fold change ≥2 and adjusted *P* value <0.001).

### Protein-protein interaction (PPI) network construction and module analysis

We analyzed the functional interactions between DEGs to interrogate the downstream signaling cascades of ACK1 in NSCLC tumorigenesis. A PPI network was generated using the STRING (http://string-db.org) (version 10.0) online database [32], with a minimum required interaction score. For further analysis, the network was imported into Cytoscape (version 3.4.0), which is an open-source bioinformatics software platform used to visualize molecular interaction networks [33].

### Identification of hub genes and analysis

The hub genes were defined as molecules with degrees ≥10, using cytohubba, a Cytoscape plug-in. Hub genes were subjected to the biological process analysis and visualized using the Biological Networks Gene Ontology tool (BiNGO) plug-in (version 3.0.3) of the Cytoscape [33]. Unsupervised hierarchical clustering of 57 hub genes was constructed using R software. PCA was used to study the expression patterns of the 57 hub genes in normal tissues and lung cancer in TCGA cohorts using the gmodels package for R. The overall survival and disease-free survival analyses of the *ACK1* genes in the TCGA lung cohort were performed using the Kaplan-Meier curve (http://gepia.cancer-pku.cn/).

### GSEA for exploration of ACK1-mediated signaling pathway

The gene expression matrices of 1,457 cell lines were downloaded from the CCLE website (https://portals.broadinstitute.org/ccle). R software was used to extract a dataset of 188 lung cancer cell lines and to process the data. The average *ACK1* expression level was used as a cutoff value to divide lung cancer cells into the *ACK1*^high^ and *ACK1*^low^ groups. GSEA was carried out in the two groups.

### Identification of the most significant module from DEGs

The MCODE plug-in (version 1.4.2) of Cytoscape was designed to gather the PPI network derived from topology to discover densely bonded molecules [33]. The PPI networks were first recapitulated using Cytoscape, and the most significant module in the PPI networks was detected using MCODE. The following criteria were applied: MCODE scores >5, degree cutoff=2, node score cutoff=0.2, max depth=100, and k-score=2. A network of the genes in the most significant module and their coexpressed genes was analyzed using the cBioPortal online platform (http://www.cbioportal.org). We also conducted the KEGG and GO analyses of genes in this module using the WEB-based Gene Set AnaLysis Toolkit (http://www.webgestalt.org/) [34, 35].

### Assessment of prognostic values of gene expression profiles

In brief, the prognostic index (PI), i.e., the risk score, was computed for each case, using the following formula: PI= *β_1_x_1_*+*β_2_x_2_*+...+*β_p_x_p_* [16], where *x_i_* and *βi* respectively represent the gene expression value and risk coefficient derived from the Cox fitting mode [16]. Cases were ranked according to risk scores, and a cutoff risk score was used to separate cases into two subgroups with distinct survival. The risk scores were calculated for the TCGA and GEO datasets using R software or an online tool SurvExpress (http://bioinformatica.mty.itesm.mx:8080/Biomatec/SurvivaX.jsp) [16]. The log-rank test was employed to check the significant difference in the survival between subgroups. The nomogram was developed with the rms package for R.

### Statistics

One-way ANOVA was adopted to compare the differences in cell survival among groups treated with different drugs. Kaplan-Meier survival curves were plotted to describe the survival status of lung cancer patients with time. A log-rank test was used to check the significance between groups. All statistics were performed with IBM ^®^ SPSS version 24 or R software version 3.6.1. A *P* value of less than 0.05 was considered statistically significant.

### Availability of data and material

The TCGA-LUAD and LUSC datasets were obtained from the TCGA portal (https://portal.gdc.cancer.gov/). Gene expression matrixes of lung cancer cell lines were obtained from the CCLE website (https://portals.broadinstitute.org/ccle). Other data are available upon request.

## Supplementary Material

Supplementary Figures
