# Supplementary material for: Identification of downstream signaling cascades of ACK1 and prognostic classifiers in non-small cell lung cancer
Source: Aging (Albany NY). 2021 Jan 20;13(3):4482–502. doi: 10.18632/aging.202408 (PMC7906148; doi:10.18632/aging.202408)
Supplement: Supplementary Figures [file aging-13-202408-s001.pdf]

SUPPLEMENTARY FIGURES

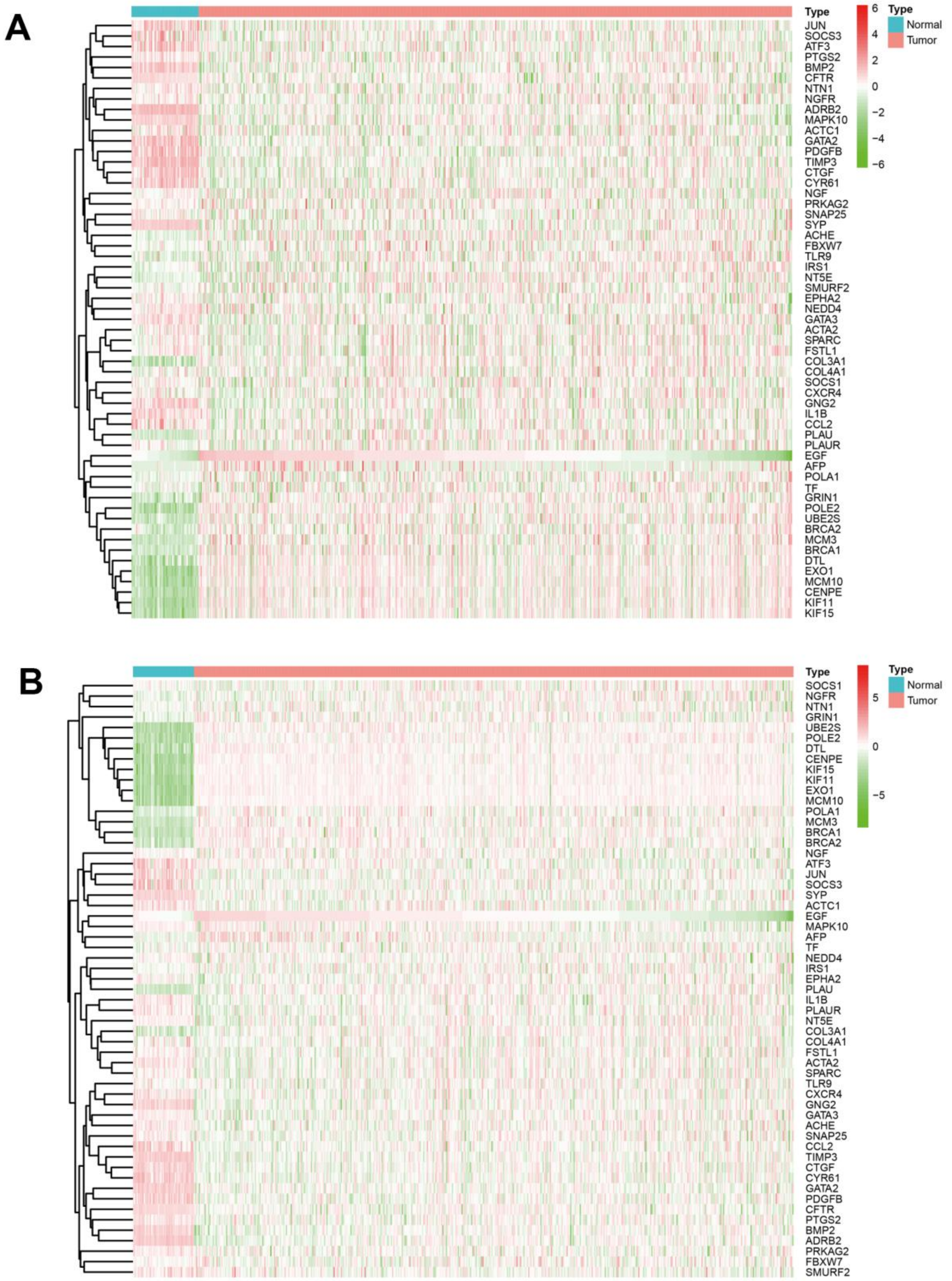

Supplementary Figure 1. Expression profiles of 57 hub genes with degree ≥20 in LUAD (**A**) and LUSC (**B**).

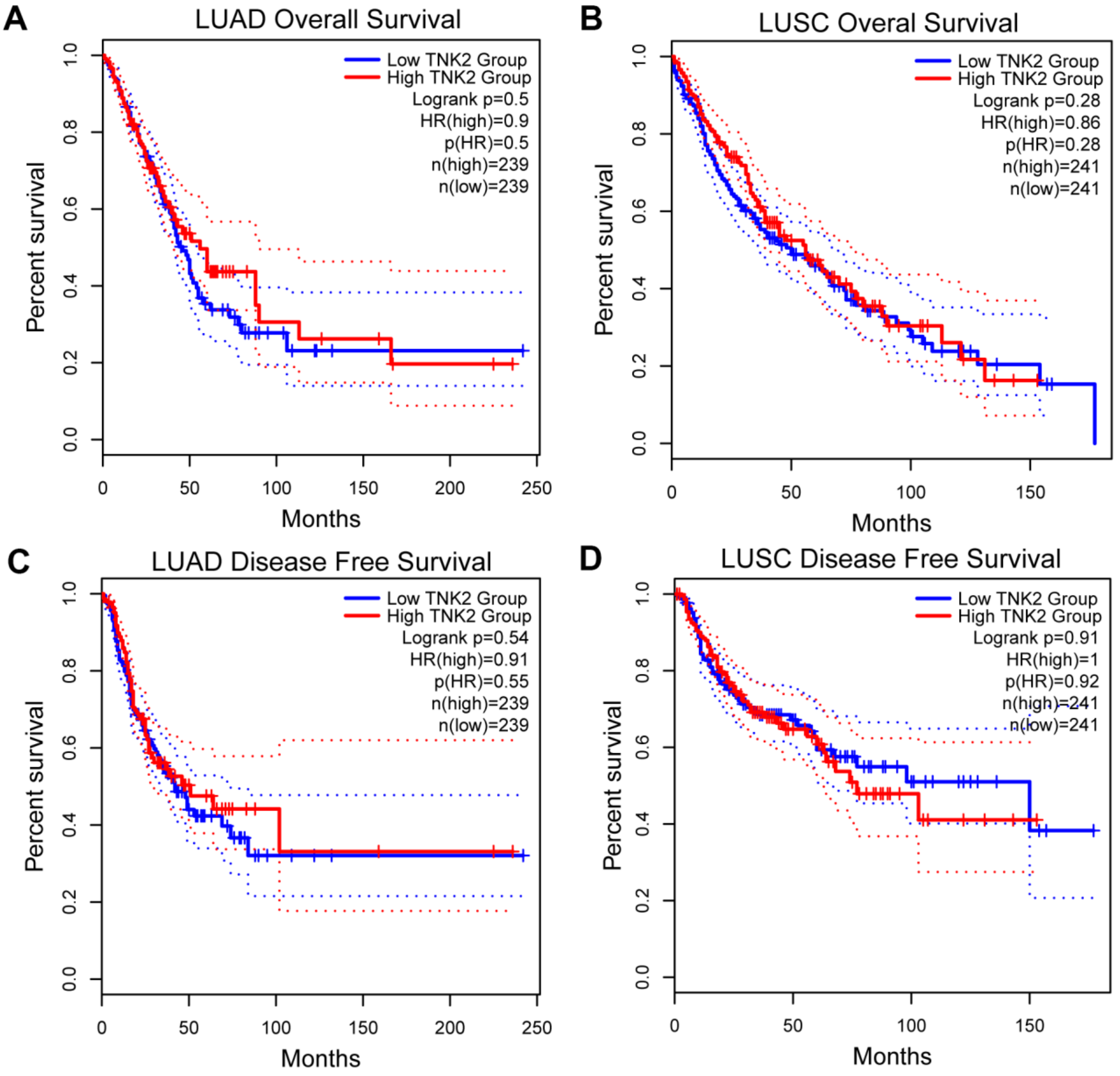

Supplementary Figure 2. Kaplan-Meier survival analysis for estimating the prognostic capacity of the *ACK1* in TCGA lung cohorts. Risk group stratification by the *ACK1* expression levels with respect to the overall survival of LUAD (**A**) and LUSC (**B**). Risk group stratification by the *ACK1* expression levels with respect to disease-free survival of LUAD (**C**) and LUSC (**D**).
